# Immunity in Atherosclerosis: Focusing on T and B Cells

**DOI:** 10.3390/ijms22168379

**Published:** 2021-08-04

**Authors:** Anastasia V. Poznyak, Evgeny E. Bezsonov, Tatyana V. Popkova, Antonina V. Starodubova, Alexander N. Orekhov

**Affiliations:** 1 Institute for Atherosclerosis Research, Skolkovo Innovative Center, 121609 Moscow, Russia; 2 Laboratory of Cellular and Molecular Pathology of Cardiovascular System, Institute of Human Morphology, 3 Tsyurupa Street, 117418 Moscow, Russia; evgeny.bezsonov@gmail.com; 3 Laboratory of Angiopathology, Institute of General Pathology and Pathophysiology, 8 Baltiiskaya Street, 125315 Moscow, Russia; 4 V.A. Nasonova Institute of Rheumatology, 34A Kashirskoye Shosse, 115522 Moscow, Russia; popkovatv@mail.ru; 5 Federal Research Centre for Nutrition, Biotechnology and Food Safety, 2/14 Ustinsky Passage, 109240 Moscow, Russia; avs.ion@yandex.ru; 6 Medical Faculty, Pirogov Russian National Research Medical University, 1 Ostrovitianov Street, 117997 Moscow, Russia

**Keywords:** atherosclerosis, CVD, immunity, T cells, B cells

## Abstract

Atherosclerosis is the major cause of the development of cardiovascular disease, which, in turn, is one of the leading causes of mortality worldwide. From the point of view of pathogenesis, atherosclerosis is an extremely complex disease. A huge variety of processes, such as violation of mitophagy, oxidative stress, damage to the endothelium, and others, are involved in atherogenesis; however, the main components of atherogenesis are considered to be inflammation and alterations of lipid metabolism. In this review, we want to focus on inflammation, and more specifically on the cellular elements of adaptive immunity, T and B cells. It is known that various T cells are widely represented directly in atherosclerotic plaques, while B cells can be found, for example, in the adventitia layer. Of course, such widespread and well-studied cells have attracted attention as potential therapeutic targets for the treatment of atherosclerosis. Various approaches have been developed and tested for their efficacy.

## 1. Introduction

Cardiovascular disease (CVD) is the most widespread cause of death. In 2013, 7.3 million people died because of CVD, which, according to world statistics, is 31.5% of total deaths [1]. To maintain of cardiovascular system health, it is recommended to stop smoking, increase physical activity, control the body mass by adopting a healthy diet, monitor blood pressure, and conserve normal levels of blood lipids and glycemia [2]. Thus, the most important thing is the diet, which can provide a good cardiovascular health status. This type of diet is related to a balanced energy intake. This involves product consumption of whole-grain foods, legumes, seafood, fish, and an increased amount of vegetables and fruits; it also involves a lower consumption of processed foods, red meat, sugar-containing foods and drinks, and refined grains [3].

Atherosclerosis is a chronic systemic inflammatory disease that attacks artery walls because of an altered inflammatory response. The development of atherosclerosis is often caused by lipid metabolism impairments [4]. Cholesterol-rich lipoproteins with apolipoprotein B are receptive to absorption and merging into the subendothelial matrix of the arteries. Due to oxidation, enzymatic and non-enzymatic cleavage, as well as aggregation, the lipoproteins contained in this matrix create pro-inflammatory particles and trigger the overlying endothelium. Then, the monocyte-derived cells internalize the subendothelium and trigger the immune response. These cells turn into mononuclear phagocytes, which absorb normal cells and altered lipoproteins, then convert into cholesterol foam cells. By remaining in the plaque, the cholesterol foam cells absorb lipids and stimulate the progression of the disease, developing a chronic inflammatory response [5].

Foam cells are considered a hallmark of atherosclerosis. At the same stage, when they are formed, a series of complex inflammatory cascades are induced. This stimulates the development of atherosclerotic lesions and leads to plaque rupture and related cardiovascular events.

Macrophages and monocytes are part of the innate immune system, which plays an essential role in the preservation of immune homeostasis by eliminating infectious agents and stimulating tissue damage repair. In atherosclerosis, these cells participate in the chronic inflammatory process, which typically occurs within the arterial wall [6].

Adaptive immunity is a very exact lifelong immune response. It is essential for distinguishing foreign- from self-antigens. The main cellular elements of adaptive immunity are T and B cells, which recognize antigens via a specific T-cell receptor (TCR) and B-cell receptor (BCR).

The set of membrane and intracellular markers underlies the classification of T cells. They express the αβ or γδ TCR, CD3, and one of the coreceptors CD4 or CD8. The TCR-CD3 complex recognizes antigens presented in the context of major histocompatibility complex molecules (MHC or human leukocyte antigen (HLA) in humans) by an antigen-presenting cell.

**Figure 1:**
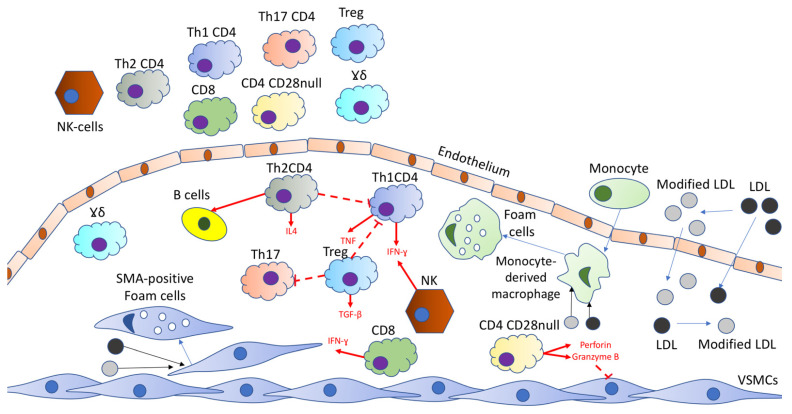
Various types of T cells are attracted to the atherosclerotic lesion site by the chemokines released by the activated endothelial cells. IFNγ (interferon-gamma) is produced by Th1 cells and has proatherogenic features. It inhibits the proliferation of smooth muscle cells, decreases collagen production, and activates macrophages. IL-4 (interleukin-4) is produced by Th2 cells and has atheroprotective effects mediated by the ability to inhibit Th1 cells. Granzyme B and perforin, which are released by CD4^+^CD28nullT-cells can damage vascular wall cells. CD8^+^ T-cells produce proatherogenic IFNγ or cause an atheroprotective effect via decreasing macrophage content in the plaque. Treg cells release TGF-β (transforming growth factor β), contributing to Th1 and Th17 response inhibition, as well as enhancing smooth muscle cell proliferation. NK-T cells are potentially involved in the destabilization of atherosclerotic plaques. No exact roles for Th17 and γδ T-cells, which are present in plaque, have yet been established.

B cells are classified in accordance with the expression of the cell-lineage marker CD19, a range of surface and intracellular proteins, their distinct B cell receptors, and their production of antibodies. B cells can also produce cytokines and, moreover, act as antigen-presenting cells. Antigen-presenting cells can present antigens to cognate naïve CD4^+^ and CD8^+^ T cells. Among such antigens, there are self- and non-self-antigens, for example, HSP60 (heat shock protein 60) and modified LDL particles. Being activated, CD8^+^ T cells proliferate and differentiate into CD8^+^ cytotoxic T lymphocytes (CTL), and CD4^+^ T cells proliferate and differentiate into specialized effector T helper (Th) cells. Naïve CD4^+^ T cells have the potential to differentiate into various cell subsets, such as effector T cells (T helper 1 (Th1), Th2, and Th17) and regulatory T cells (Treg). The type of antigen, T-cell receptor signal intensity, and the local cytokine environment define the Th subsets into which T-cell can differentiate. These factors mediate Th polarization in atherosclerotic lesions [7]. The implication of immune cells in atherogenesis is briefly summarized in Figure 1.

## 2. T-Cells

Studies conducted in the 1980s gave rise to the assumption that adaptive immunity plays an important role in human atherosclerosis. These studies demonstrated broad expression of the MHC-II molecule linked with human leukocyte antigen D (HLA-DR) in human atheroma, alongside a multiplicity of CD3^+^ T cells [8]. The majority of the T cells found in human atherosclerotic plaques show an effector memory phenotype. Most of them display an activation token and approximately 2/3 are CD4^+^ T helper cells (Th) carrying the αβ T cell receptor (TCR) [9].

Human atherosclerotic plaques also contain a multitude of CD8^+^ cytotoxic T cells. Among others, the first cells accumulating in atheroma are T cells, which are fortified with unstable plaques. Since atherosclerotic plaques tend to rupture, this might lead to blood-clotting, blood vessel blockage, and acute cardiovascular events [10]. Using monoclonal TCR, such as HLA-DR^+^ and CD28^null^ T cells, it was found that atherosclerotic lesions in patients, who suffer from acute coronary syndrome (ACS), are not only subjected to macrophage infiltration, but are also infiltrated by oligoclonal T cells, displaying a constant, antigen-driven immune response, and specific activated subsets of T cells [11].

It has already been noted that the identification of the corresponding antigens that are recognized by these cells remains the primary unanswered question. While the plaque inflammatory environment is able to recruit a normal heterogeneous polyclonal cell population, most of the T cells that are insulated and cloned from human plaques react to oxidized low-density lipoprotein (oxLDL) or other antigens (i.e., antigenic determinant of the bacterial wall, in an HLA-DR-restricted manner) [12]. In 78% of patients with acute myocardial infarction, the DNA of oral viridian streptococci was found in their blood clots. This indicates that in acute coronary syndrome, the activation of inflammatory pathways is not limited to coronary vessel damage and may also be contained in a thrombus [13].

A fairly large number of antigens are able to be detected systematically. Therefore, effector T-cell reactions, in most cases, appear in secondary lymphoid organs such as the lymph nodes and spleen. It is generally assumed that activated T cells circulate in atherosclerotic lesions, where the putative antigens are settled. This hypothesis is confirmed by the identification of an elevated number of chemokine receptors participating in the mobilization of T-cell plaques. One such example is the presence of CCR5 and CXCR3 on the surface of CD4^+^ T cells in patients with CAD [14]. It is noteworthy that the suppression of chemokine receptors (CCR5 and CXCR3) participating in the recruitment of T cells, circulating into the human plaque, weakens the development of atherosclerotic lesions in animal models [15]. Thus, atherogenic T-cell responses are possibly characterized as systemic. This is an argument for studying T-cells both inside the plaque and systemically, by analyzing the profile of T-cells in peripheral blood [11].

### 2.1. T Cells within Plaque

Atherosclerotic plaques store plenty of CD4^+^ T cells. In reaction to comprehensive stimulation with antigen, co-stimulators, and peculiar cytokines, T cells separate distinct effector or Th subsets which are differentiated by the cytokines that they secrete [16]. The main characteristic subsets of Th are: (1) Th1, which secretes interferon (IFN)-γ; (2) Th2, which secretes IL-4, IL-5 and IL-13; and (3) Th17, which secretes IL-17 and IL-22. Chronic or secondary exposure to the antigen that presents during atherosclerosis usually occurs in a predominant subset of Treg. Significant arguments point to the importance of Th1 and IFN-γ in atherosclerosis development and inflammation [17].

In human atherosclerotic lesions, the greatest signs of activity are shown by Th1, which is the most common subtype of T cells [18]. For example, they secrete IFN-γ, TNF-α, and IL-2 [19]. It is also important to note that these cells secrete IFN-γ when stimulated by oxidized LDL and LDL. It has been detected that IFN-γ generates the progression and enhances atherosclerotic lesion resistance in various ways, entailing changes in endothelial function, recruitment of inflammatory cells in the lesion, and intervention in the export of cholesterol from cells in the lesion [20]. One of the key cytokines of the Th2 subset, IL-4, significantly suppresses Th1 differentiation and follows IFN-γ secretion, which indicates a potential protective role against atherosclerosis. However, definitive pathological evidence in humans is currently lacking [21].

An interaction between variants near the IL-5 gene locus and coronary artery disease has been shown [22]. This allows us to guess the role of the Th2 subset in modulating CAD development and promotion. A possible geroprotective effect has been proposed for IL-5, due to its negative correlation with the density of the carotid intima medium (IMT), a marker of subclinical atherosclerosis [23]. It is believed that subsets of Th17 are linked with atherosclerosis [24]. However, their role is unconfirmed. Studies have also shown that the development of IL-17A caused by damaged T cells is linked with inflammation and plaque destabilization [25]. In humans, a subset of CD28_null_ CD4^+^ T cells is enlarged against a background of inflammatory disease, cytomegalovirus infection, and old age. By producing anti-inflammatory cytokines, which include IFN-γ, these cells demonstrate cytotoxicity [26].

Clonally expanded CD28_null_ CD4 T-cells, which are able to cause and enhance inflammation, were found in unstable coronary plaques [27]. Most of these cells uniquely identify HSP60 and express members of the TNF receptor family. Such an example is OX40 (CD 131), which is able to act as an alternative co-stimulating receptor [28]. Moreover, the cells are shown to be regulatory T cells which are stable in the context of in vitro suppression [29]. They also include a number of CD4^+^ T cell subsets that are able to suppress immunity and are essential in self-tolerance and patronage, despite autoimmunity.

About 1–5% of all T cells in atherosclerotic lesions are T_regs_, which is less than 25% of their presence in other chronically inflamed tissues [30]. The results of some studies have shown a decrease in the amount of T_regs_ in unstable plaques [31], implying an atheroprotective role that utilizes their anti-inflammatory function as an immune regulator. Other studies offer a different point of view, indicating T_regs_ content elevation in lesions [32]. This may be due to both an adjustment of the functional state of T_regs_ and its compensatory elevation to balance the level of T-cell activity in the plaque.

According to Klingenberg and colleagues’ recent report, an elevation of the amount of Tregs in coronary artery clot aspirate was found in 16 patients with ACS, in contrast to circulating Treg in the same patients or healthy control groups [33]. Remarkably, the T cells demonstrated limited TCN expression; thus, these data prove differential, antigenic trapping of Tregs in the thrombus as a result of ACS [34].

As a rule, in human atherosclerotic plaques, CD8+ cytotoxic T cells are not as common as CD4^+^. However, in severe lesions, they may comprise no more than 50% of the cells, which indicates a possible role in plaque inflammation and instability [35].

NK-T—Natural Killer T cells—is a precise subset of T cells expressing both natural killer and T cell markers. Stimulation by lipid antigens, which is mediated via the MHC-I-like CD1d molecule, activates NK-T; this is of additional interest in the study of atherosclerosis [36]. The cells that are contained in the human atheroma exhibit CD1 and NK-T cells, which indicates the proatherogenic role of this subtype of T cells [11].

### 2.2. Circulating T Cell Subpopulations

In patients with coronary artery disease, a lymphocyte subpopulation profile in the thoracic lymph nodes appeared to differ from the blood profile. This includes a higher share of B cells, a lower share of CD8^+^ T cells, a twice higher CD4/CD8 ratio, and CD4^+^CD69^+^ cell saturation, as well as T_regs_ [37,38]. In the peripheral blood of patients with cardiovascular diseases a higher share of effector memory T cells (TEM cells or TEM) characterized as CD3^+^CD4^+^CD45RA^−^CD45RO^+^CCR7^−^ were found, which relate to the degree of atherosclerotic lesions in the coronary and brain carotid regions [39]. In comparison with these results, other studies have demonstrated a CCR7^−^ T cell elevation in patients with coronary artery disease [40], and T cell memory increase in patients with subclinical carotid atherosclerosis [41].

TEM appeared as a subset of T cells with the strongest bond with atherosclerosis in carotid and coronary vessels at various disease phases. An essential correlation was spotted between TEM and total plasma cholesterol, as well as LDL cholesterol. Despite the link between TEM and carotid artery atherosclerosis, there was no dependence on the classical cardiovascular risk factors, which proves the validity of the adaptive immune response in cardiovascular disorders [42]. After the removal of the antigen that triggered the immune response, TEM cells and central memory T cells (TCM) are stored in the memory pool. They store the memory of (1) antigen specificity, (2) the whole range of cytokines they have produced and (3) the site where their effector function is required. With repeated exposure to the TEM antigen in inflamed peripheral tissues (in this particular case an atherosclerotic plaque), it quickly shows effector effects. This is mainly due to the CCR5 and CXCR3 expression [43].

HLA-DR expression is a sign of effector function, and some studies have shown an elevation in activated HLA-DR^+^ T cells in patients with coronary heart disease [44]. It was found that Th1 cells are more common in the blood of patients suffering from acute coronary syndrome [45]. However, it remains to be revealed whether this represents an acute reaction to myocardial injury or an underlying CAD. It was also found that a subset of INF-γ-secreting Th17 cells, specifically Th1/Th17 cells, is caused by the progression of ACS, which proves the significance of IFN-γ in atherosclerosis [11].

Although Th17 was linked with a high-risk level of cardiovascular disease, this association turned out to be inconsistent [24]. Recent studies have shown a negative correlation between circulating Th2 cells, the thickness of the common medium carotid intima (IMT), and the cardiovascular events threat, as well as a negative bond between the number of Th1 cells and the progression of complications associated with atherosclerosis [46]. Over the ACS period, the traditional immunological synapse mediated by antigen–TCR involvement (signal 1) and co-stimulatory receptors such as CD28 (signal 2) is the circulation of unsound T cells [47].

Essentially, CD3^+^CD4^+^TCR zeta-dim, a subset of T cells with reduced levels of the TCR zeta subunit, also referred to as CD247, binds the involved TCR-CD3 complex to downstream intracellular signal transduction pathways. Patients with ACS have been shown to have higher levels of CD4^+^CD28^null^ T cells [48]. Notably, an association was found between higher circulating levels of CD4^+^CD28^null^ cells and a poor prognosis in ACS relapse. TCR zeta and CD28 chain regulation lowering usually happen following antigen involvement or in reaction to inflammatory stimuli as a feedback mechanism aimed at setting the immune response [11].

Whereas intact TCR signaling is highly important for upholding immune homeostasis through the generation and functioning of regulatory T cells subsets, changes in signal pathways can lead to an increase in TCR zeta-dim T cells. These are able to weaken modulator feedback signals, thereby potentially limiting the sensitivity of CD4^+^CD28^null^ T cells to suppression. TCR zeta-dim T cells and CD4^+^CD28null T cells are able to react to stimuli isolated from the antigen-mediated TCR pathway [49]. In addition, human-circulating or intraplaque CD4^+^CD28^null^ T cells from patients with ACS exhibit IL-12 receptors even when antigen stimulation is deficient. This increases the expression of the CCR5 chemokine receptor CD161 and the C-type lectin receptor CD161, which are involved in the regulation of the tissue homing of effector T cells after IL-12 stimulation. Therefore, CD4^+^CD28null T cells could function like NK cells with anti-inflammatory activity, even in the irreconcilable state of enlarged tissue trafficking and homing after an IL-12-inducing host infection connected with accrual in inflammatory lesions. Therefore, it is generally assumed that both antigen-dependent and autonomous mechanisms are crucial for obtaining responses in subsets of memory T cells with CD28 and/or TCR zeta-chain defects, thus contributing to the pro-inflammatory and pro-atherosclerotic response [50].

During atherogenesis, adaptive immunity has both stimulating and suppressive effects on plaques [51]. As for the anti-inflammatory or anti-atherosclerotic side of T-cell function, analysis of circulating Tregs gave contrary results. ACS patients have lower levels of CD4^+^CD25^+^forkhead box protein 3 (FoxP3^+^) circulating in T cells, and T_regs_ detached from the blood of the same patients showed a lowered ability to suppress oxLDL-induced CD4^+^CD25 proliferation [31].

However, in patients with stable CAD, no significant association with the spread of atherosclerotic disease was detected. No link between the stable degree and progression of CAD and the levels of circulating T_regs_, designated as CD4^+^CD25^hi^CD127^lo^, was shown. This proved an association between an ST-elevation myocardial infarction (STEMI) T and a high level of Tregs [33].

More distinctly, inflammatory activation in ST-elevation myocardial infarction (STEMI), confirmed by elevated IL-6, is able to explain the proportional compensatory balance of Treg, similar to the observed elevation in IL-10 [52].

On the contrary, patients with acute coronary syndrome have shown a decrease in level of Tregs circulating without elevation of ST levels [33]. Ultimately, since CCR5 not only controls effector T cells but also directs Tregs and moves them into inflamed non-lymphoid tissue, assuming that CCR5^+^ T_regs_ constitute a subgroup of ‘effector’ T_regs_ cells, the levels of circulating CCR5^+^ Tregs were evaluated in both subclinical carotid artery patients and patients with CAD. The tricky role of T_regs_ in atherosclerosis is currently being studied. This is proved by new data on the atheroprotective role of this subset of T cells, as acquired in mouse models [53].

## 3. B-Cells

T cells’ role in atherosclerosis has been studied for decades, but B cells have only recently become an object of curiosity. Hints of B cell involvement in the pathogenesis of the atherosclerotic lesions were observed during animal studies [54]. However, lately, such hints also began to appear in humans. Along with the whole blood gene expression profiles of Framingham Heart Study participants, a network integrative data analysis of genome-wide linkage studies showed the presence of B-cell immune responses as provocative factors for coronary artery disease [55].

Unlike T cells, just a small amount of B cells are able to be found locally in atheroma. At the same time, a multitude of B cells could be detected in the atherosclerotic vessels adventitial layer, where they exhibit a structural organization close to the tertiary lymphoid organ, which is associated with the presence of a chronic immune response [56].

It was found that, in atherosclerotic lesions, B cells are oligoclonal and undergo antigenic proliferation [11]. Being Th cell-dependent, the antigen-driven B-cell response slows down and leads to the formation of high-affinity antibodies which are exposed to class switching.

The whole process happens in structures of special purpose inside the lymphoid organs—the germinal centers. It has been shown that a specific subset of Th cells or T-follicular helper cells (Tfh) is responsible for the location of the germ center, as well as for supplying B cells with the assistance necessary for the proliferation and maturation of affinity [57]. It was established that the Tfh cells do not express fewer cytokines; they also provide a diversity of surface receptors, such as CD40L (CD154) or OX-40, in comparison to other subsets of Th cells [14]. Research on the subject of Tfh cells and their role in atherosclerosis is still ongoing. The B cells liable for this type of response emerge from the bone marrow and are referred to as B2 cells [58]. The antibodies secreted by B cells include all classes of human immunoglobulins (Ig), i.e., IgM, IgG, IgE, and IgA. In the blood serum of patients with atherosclerosis, IgG antibodies directed against oxidation-specific epitopes can easily be found (in particular, the aldehyde-modified peptide sequences of apolipoprotein B-100) [59]. It has also been revealed that self-reactive IgG against transhelin (TAGLN), a cytoskeletal protein, is secreted by B2 cells located in carotid artery plaques [60]. It is noteworthy that these antibodies cross-react with the antigenic determinants of the bacterial wall of gram-negative bacteria related to the *Enterobacteriaceae* family, which again indicates the infection’s possible role in atherosclerosis promotion [61]. Obviously, additional studies are being conducted to further understand the role of the connection with the cardiovascular risk of IgG and IgM against oxidation-specific epitopes (OSEs) and other antigens that can be detected in atherosclerotic plaques. Experimental studies have revealed that, in addition to the production of atherogenic antibodies in B2 cells, they can aggravate atherogenesis. This is due to antibody-independent mechanisms that enhance the effect of pro-inflammatory cytokines [62].

Immunoglobulin IgA is able to be detected on mucous membrane surfaces, where it contributes to the major defense line against pathogens at reduced levels of concentration within the circulation. Despite the lack of data regarding the role of IgA in atherosclerosis, there may be a link between high serum IgA titers and progressive vascular diseases, as well as myocardial infarction. So far, no mechanism has been proposed to clarify this relationship. However, the latest information on the role of the gut microbiome in cardiovascular diseases provides new insights into the role of IgA in atherosclerosis [63].

In addition to B2 cells, a small subset of B1 cells also exist. It consists of long-lived, non-circulating cells that are mostly detected in the spleen, peritoneum, or pleural cavity [64]. These cells secrete poorly specific natural IgM antibodies, creating a rapid and T-cell-independent humoral response. Secreted B1 antibodies are polyreactive and are the main defense against pathogens. Natural IgM antibodies represent an essential ratio of IgM in noninfected humans, and up to 30% of them are targeted particularly against OSEs [65]. Some clinical studies have demonstrated that the titers of such naturally occurring oxidation-specific antibody IgMs correlate, on the contrary, with atherosclerotic load, which is estimated by carotid BMI [66], as well as with the risk of stroke and acute myocardial infarction. The atheroprotective mechanism of natural IgMs has yet to be determined. However, some experimental studies have demonstrated that these antibodies inhibit the internalization of oxLDL by macrophages and restrain the storage of apoptotic cells by enhancing efferocytosis.

## 4. T-Cell Based Therapy

Various compounds have been shown to regulate Tregs and thus to have an efficacy in the treatment of atherosclerosis in animal models. Data on several of the most investigated drugs are summarized in Table 1. Therefore, pharmacological regulation of the numbers and immunosuppressive activity of Tregs may provide valuable treatment options for atherosclerotic diseases.

Atherosclerosis treatment options involving antibodies and cytokines are of particular interest. IL-2 stimulates the proliferation and differentiation of T cells and Treg effectors. However, low doses of IL-2 give a strong result in the atherosclerosis treatment, due to the selective expansion of Tregs with essential sensitivity to IL-2. This method was, for example, used in the clinical treatment of systemic lupus erythematosus [75]. Administration of antibodies (both oral and intravenous) against CD3 suppresses atherogenesis and the possible development of atherosclerotic plaques, causing T_regs_ expansion and decreases the number of CD4^+^ T cells in mice [76]. Successful suppression of atherosclerosis is also possible with treatment using anti-CD3 antibodies and the IL-2 complex [76]. Integrin αvβ8 mediates TGF-β activation. Thereby, manipulation of the avß8 integrin is able to modulate Trg function to interrupt atherosclerotic disease mediated by effector T cells [77]. G-CSF (granulocyte colony-stimulating factor) modifies immunity and enhances immune diseases in animals, elevates the amount of Tregs and IL-10, and lowers IFN-γ levels in ApoE^−/−^ mice [78].

In atherosclerosis, physical therapy is able to play a protective role. As an illustration, ultraviolet B radiation weakens the development of atherosclerosis in mice that are inclined to atherosclerosis, due to the increased function of Tregs and the regulation of the effector response of T cells [79].

## 5. B-Cell Based Therapy

### 5.1. Rituximab

It is widely thought that rituximab eliminates B-lymphocytes; potentially through complement-dependent and antibody-dependent cell cytotoxicity and induction of B-cell apoptosis. Recent studies examining the influence of rituximab on the cardiovascular system are not extensive. Hypothetically, selective suppression of the effect of B2 cells on the vascular wall of patients with rheumatoid arthritis can be the endpoint of endothelial dysfunction and atherosclerosis prevention. A couple of experimental reports have been published on the positive effect of rituximab on the lipid profile and early markers of atherosclerosis (enhancement of endothelial function) in patients with atherosclerosis [80].

Therefore, several findings suggest that, in incidents of productive lowering of rheumatoid arthritis activity, a lower index of atherogenicity and improved endothelial function are found. However, the rituximab effect on the promotion of atherosclerosis, and the corresponding cardiovascular events in patients with rheumatoid arthritis, requires further study [81,82].

It is assumed that statin therapy is a long-term strategy for primary and secondary prevention of cardiovascular diseases [83]. A five year follow-up showed that the high-dose administration of statins, such as atorvastatin and simvastatin, is able to cause commensurable cardioprotective and hypolipidemic effects both in patients with rheumatoid arthritis and in the general population. Even though, at the initial stage, patients with rheumatoid arthritis had a lower total cholesterol level, it has been established that statin usage suspension for more than three months in patients with rheumatoid arthritis is caused by an elevated risk of myocardial infarction by 67% [84].

DREAM—the Dutch Rheumatoid Arthritis Monitoring study, provided evidence of a reduction in the effect of antiretroviral therapy while using concomitantly with statins. After six months, patients with rheumatoid arthritis who received combined statins and rituximab (*n* = 23) had a higher DAS28 score, in contrast to patients who did not take statins (control group, *n* = 64), adjusted for gender, baseline DAS28 level, and rheumatoid factor positivity [85]. Compared to the control group, the period of usefulness of rituximab in patients receiving statins was shorter—seven months as opposed to nine months. This study points to the need for limiting the concomitant treatment with statins and rituximab in patients with rheumatoid arthritis. Treatment of patients suffering from lymphoma gave the opposite results. For almost four years, the use of antiretroviral therapy and statins equally did not have a negative impact on clinical outcomes. Statins’ influence on the effectiveness of rituximab is a clinical problem, and confirmation of its prognostic importance requires further studies [86].

### 5.2. Modulating B-Cell Receptor Signaling

BCR signaling plays a significant role in the control of B cell activation, proliferation, and differentiation. Therefore, strict regulation by costimulatory receptors is required. Ibrutinib is used for cancer therapy and suppresses Bruton tyrosine kinase (Btk) below the BCR. As another option, BCR signaling can be negatively regulated by using mAb epratuzumab, which activates the CD22 inhibitory receptor. Originally, Epratuzumab was created to treat systemic red lupus [87]. Currently, there is no preclinical data on the significance of these factors in the formation of atherosclerosis. Since the specificity of BCR and the power of the BCR signal are crucial for determining decisions about the fate of B cell development, drugs modulating the BCR signal are able to affect the distribution of subsets of B cells, which in turn is able to affect atherogenesis. We recently demonstrated that low-dose treatment with ibrutinib leads to margin zone lowering and is linked with an increase in the number of FOB cells [88]. Therefore, it seems that powerful BCR signaling promotes the development of marginal zone (MZ) B cells. Therapeutic modulation of BCR signaling is able to guide the differentiation of B2 cells towards the atheroprotective B cell fate. It has also recently been reported that MZB cells have thermal protective activity. Using a genetic model of MZB cell deficiency, it has been shown that the role of MZB in the negative regulation of proatherogenic TFH cells, via the PDL1 (programmed cell death ligand 1) axis, programmes cell death 1 [89]. Before this, it was assumed that a potential atheroprotective role might appear through the secretion of OSE-specific antibodies. However, the contribution of B cells could not be excluded. It is interesting to study the additional effect of BSR signaling on subsets of B cells and atherosclerosis in patients undergoing BSR-modulating therapy. Clinically, ibrutinib is linked with a high risk of atrial fibrillation and hypertension [90].

### 5.3. Targeting B-Cell Costimulation and Immune Checkpoint Inhibitors

The synergy between B and T cells is vital for adaptive immunity. B cells provide antigens and send costimulatory signals to T cells. The two most notable methods have been extensively investigated in experimental atherosclerosis: CD40-CD40L dyad and CD80/CD86-CD28/ CTLA4 system (cytotoxic T-lymphocyte associated protein). Preclinical studies of CD40 or CD40L deficiency indicate predominantly proatherogenic functions of this dyad [91]. However, it has been hypothesized that T-cell and CD40-dependent B-cell responses are proatherogenic circulating CD40^+^ B cells correlated with a reduced risk of stroke, which may be related to the importance of CD40 in Breg differentiation. Although CD40/CD40L inhibitors have a powerful anti-inflammatory effect in preclinical studies, their clinical use has been complicated by severe side effects. CD80 and CD86 on B cells interact with both the CD28 costimulatory receptor and the CTLA4 inhibitory receptor on T cells [92].

Since CD86 generates the induction of Th1 immunity, experimental studies suggest a predominantly proatherogenic function of CD80/CD86,75,76. At the same time, CD86^+^ B cell levels correlate with the degree of stenosis and the frequency of stroke in humans. However, CD80/CD86 signaling is also potentially required for the induction of atheroprotective regulatory T cells. For the treatment of rheumatoid arthritis, the CTLA4Ig (abatacept) design was approved, preventing CD80/CD86 from interacting with CD28 and activating T cells. Despite the positive effect of CVD on cardiovascular parameters in preclinical studies, cardiovascular parameters have not been evaluated in clinical trials [93].

In contrast, many cell types, which include antigen-presenting cells and tumor cells, express T-cell-inhibiting ligands. They target immune checkpoint inhibitors (ICIs), which are often used in cancer therapy. ISCs are clinically approved; they target programmed cell death 1 (nivolumab, pembrolizumab), PD L1 (atezolizumab, durvalumab, and avelumab), and CTLA4 (ipilimumab). However, they have potentially detrimental effects on cardiovascular disease, and cardiovascular adverse events have been reported [94].

Interestingly, high T cell activity after checkpoint suppression could also contribute to T cell-mediated effects on B cells. Elevated B cell activation and plasmablast levels have been reported in patients treated with immune checkpoint inhibition (ICI), which carries an increased risk of immune-related side effects. Even though ICI has possible proatherogenic effects, most likely through its effect on T cells, consequential effects on B cells should not be missed [95].

## 6. Conclusions

Atherosclerosis is the leading contributor to mortality rates worldwide. The most important components of the pathogenesis of atherosclerosis are, without doubt, lipid metabolism alterations and inflammation. When considering inflammation, the crucial role is played by immune cells, of which T and B cells must be noted. The impact of T cells is well-studied, especially in contrast to B cells, which became a topic of interest relatively recently. This may be explained by the low level of B cells in the atheroma itself—T cells are presented there in all their diversity.

A special research interest lies in the scope of therapeutic targeting. Various approaches have proven their effectiveness in atheroprotection via T cells, especially Tregs regulation. Such approaches include the use of well-known drugs, antibody and cytokine treatment, as well as ultraviolet B radiation. When considering B cells as a target, rituximab seems both interesting and promising.

## Figures and Tables

**Table 1:** Effects of several well-known drugs on Tregs.

Drug Name	Effects	Mechanisms	Reference
Mycophenolate mofetil	Atherosclerosis suppression	Enhancing the number of Tregs Decreasing the activation of T cells, NKs, macrophages and DCs Decreasing the MMP and cathepsin generation Stimulating the expression of lipid metabolism-associated genes	[67,68]
Rapamycin	Immunotherapy in T cell-mediated CVD	Immunosuppressive activity Triggering Treg expansion and lowering T effector cells	[69]
Orally activated vitamin D3	Atherosclerosis suppression	Enhancing Treg levels Triggering tolerogenic DCs Lowering IL-12 expression, and enhancing IL-10 expression in mice	[70,71]
Pioglitazone	Atherosclerosis suppression	Modulating the of Th1/Th2 cells balance Increasing Treg response Enhancing the SMC level and collagen content in ApoE-deficient mice	[72]
Simvastatin	Beneficial in ACS patients	Enhancing Treg levels Stimulating TGFβ, IL-10, and FOXP3 expression in the atherosclerotic plaques of ApoE^−/−^ mice Inducing Treg expansion in ACS patients	[73]
Atorvastatin	Beneficial in ACS patients	TriggeringTreg expansion Inducing FOXP3 expression in humans but not in C57BL/6 mice	[73]
Amygdalin (vitamin B17)	Atherosclerosis suppression	Modulating the lipid metabolism by lowering plasma total cholesterol (TC), triglyceride (TG), and LDL levels Triggering Treg expansion and upregulating IL-10 and TGF-β expression in ApoE^−/−^ mice	[74]
